# Coenzyme Q improves mitochondrial and muscle dysfunction caused by CUG expanded repeats in *Caenorhabditis elegans*

**DOI:** 10.1093/genetics/iyae208

**Published:** 2024-12-27

**Authors:** Joana Teixeira, Anu-Mari Harju, Alaa Othman, Ove Eriksson, Brendan J Battersby, Susana M D A Garcia

**Affiliations:** Institute of Biotechnology, HiLIFE, University of Helsinki, Helsinki 00790, Finland; Institute of Biotechnology, HiLIFE, University of Helsinki, Helsinki 00790, Finland; Institute of Molecular Systems Biology, ETH Zurich, Zurich 8093, Switzerland; Biochemistry/Developmental Biology, Faculty of Medicine, University of Helsinki, Helsinki 00014, Finland; Institute of Biotechnology, HiLIFE, University of Helsinki, Helsinki 00790, Finland; Institute of Biotechnology, HiLIFE, University of Helsinki, Helsinki 00790, Finland

**Keywords:** CUG repeats, myotonic dystrophy, RNA repeat toxicity, mitochondrial dysfunction, coenzyme Q, *Caenorhabditis elegans*

## Abstract

Expansion of nucleotide repeat sequences is associated with more than 40 human neuromuscular disorders. The different pathogenic mechanisms associated with the expression of nucleotide repeats are not well understood. We use a *Caenorhabditis elegans* model that expresses expanded CUG repeats only in cells of the body wall muscle and recapitulate muscle dysfunction and impaired organismal motility to identify the basis by which expression of RNA repeats is toxic to muscle function. Here, we performed 2 consecutive RNA interference screens and uncovered coenzyme Q metabolism and mitochondrial dysfunction as critical genetic modifiers of the motility phenotype. Furthermore, coenzyme Q supplementation reduced the toxic phenotypes, ameliorating the motility impairment and mitochondrial phenotypes. Together our data show how the expression of expanded RNA repeats can be toxic to mitochondrial homeostasis.

## Introduction

Nucleotide repeat sequences are prevalent in coding and noncoding regions of the genome (Lander *et al*. 2001). These sequences can form diverse secondary structures and thus are prone to breakage, replication stalling, and genome rearrangements that can lead the repeats to contract or expand (Brown and Freudenreich 2021). The instability in repetitive sequences is associated with expansions in more than 40 human neuromuscular disorders (Paulson 2018). Expanded repeats often exhibit a genotype–phenotype correlation that affects disease severity and age of onset (Andrew *et al*. 1993; Tokgozoglu *et al*. 1995). At the cellular level, the pathogenic mechanisms of RNA repeat toxicity are complex: transcript loss-of-function, inappropriate interaction with RNA-binding proteins (Miller *et al*. 2000), and non–AUG-dependent translation of the repeats into toxic proteins (Swinnen *et al*. 2020). RNA repeats have been described to act as pathogenic gain-of-function species by sequestering RNA-binding proteins (Miller *et al*. 2000).

The most prevalent form of adult-onset muscular dystrophy (Harper 2009), myotonic dystrophy type 1 (DM1), results from a CTG expansion in the 3′ UTR of the dystrophia myotonica-protein kinase gene (Brook *et al*. 1992; Mahadevan *et al*. 1992). DM1 is a dominantly inherited systemic disorder characterized by progressive muscular weakness, atrophy, and myotonia (Harper 2009; Chau and Kalsotra 2015). In patients, the CTG expansion ranges from 50 to 4,000 repeats, whereas healthy individuals have 5–35 repeats (Turner and Hilton-Jones 2010). Transcripts bearing CUG expansions constitute the prototype for gain-of-function RNA repeat toxicity (Ranum and Cooper 2006). Any RNA transcript bearing an expanded CUG repeat in the 3′ UTR is sufficient to induce DM1-like features (Mankodi *et al*. 2000) and can accumulate in the nucleus as discrete foci (Taneja *et al*. 1995). A few modifiers of DM1 pathogenesis have been identified, with the best-characterized corresponding to the developmentally regulated RNA-binding factor family muscleblind-like (MBNL; Miller *et al*. 2000), which is sequestered by the expanded repeats (Goodwin *et al*. 2015). Despite this, the underlying spectrum of mechanisms by which the expression of CUG RNA repeats induce cellular dysfunction and contribute to the molecular pathogenesis remains poorly understood.

To identify molecular factors that modulate the RNA toxicity of CUG repeats, we took advantage of an established *Caenorhabditis elegans* model expressing expanded CUG repeats only in the body wall muscle cells (Garcia *et al*. 2014) to perform an RNA interference (RNAi) genetic screen. This model recapitulates phenotypes characteristic of DM1, including impaired motility, accumulation of the toxic RNAs as nuclear foci, and colocalization of MBL-1, the sole ortholog of the MBNL family, with the RNAs bearing expanded repeats (Garcia *et al*. 2014). Here, we genetically establish the expression of toxic RNAs as drivers of mitochondrial dysfunction in muscle cells affecting cellular function in a DM1 model. Metabolic supplementation with coenzyme Q (CoQ) ameliorates the mitochondrial phenotypes and the movement defect associated with the expression of the expanded CUG repeats. Together, these findings point to the importance of mitochondrial dysfunction in the molecular pathogenesis of RNA expanded repeat disorders.

## Results

### Expanded CUG repeats alter CoQ cellular requirement

**Fig. 1.**
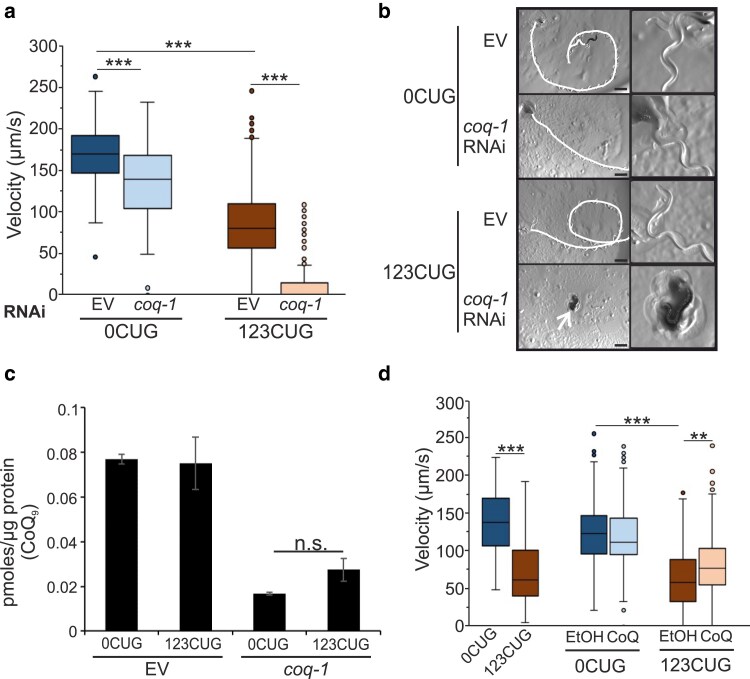
COQ-1 is a suppressor of 123CUG toxicity. a) Velocity of 0CUG and 123CUGs on EV and *coq-1* RNAi, *n* ≥ 110 animals/condition. b) Representative DIC microscopy images of 0CUG and 123CUG animals RNAi inactivated for EV or *coq-1*. White line indicates animal tracks, and arrow shows paralyzed animal. Scale bar: 1 mm. c) CoQ_9_ lipid levels of 0CUG and 123CUG animals RNAi inactivated for EV or *coq-1*. Bars = mean ± SEM. *P*-value was determined by 2-tailed Student's *t*-test. d) Velocity of 123CUG and 0CUG animals supplemented with 150 µg/mL CoQ_10_, *n* > 100 animals/condition. a and d) Motility assays: 3 independent experiments/assay. *P*-value was determined by Wilcoxon statistical test, with Bonferroni correction. **P < 0.005, ***P < 0.0001. Box: 25–75th percentile; whiskers: 1.5 × interquartile range; line in the box: median; dots: outliers.

To understand how toxic RNAs cause pathogenesis, we performed 2 consecutive RNAi screens (Supplementary Fig. 1), using *C. elegans* as a model system to identify genetic modifiers of RNA expanded CUG repeat toxicity. Previous work from our laboratory and studies by other researchers have shown that RNA metabolic pathways perform important roles as mediators of toxicity, in particular factors implicated in mRNA processing and stability of transcripts expressing GC-rich sequences, including CUG repeats (Ravel-Chapuis *et al*. 2012; Garcia *et al*. 2014). Thus, our first screen sought to enrich for potential modifiers of RNA repeat toxicity by uncovering genes involved in the regulation of RNA metabolic processes in the muscle cells. Here, we used a transgenic *C. elegans* strain expressing an RNA degradation reporter. This strain expresses a GFP construct with a GC-rich sequence in its 3′ UTR that leads to mRNA degradation (Garcia *et al*. 2014). Using this reporter, we screened 1,824 RNAi clones covering the first two-third of chromosome I to identify factors that could stabilize the GC-rich mRNA reporter and increase the GFP signal (Supplementary Fig. 1; *Materials and methods*). This first screen identified a large set of positive hits; therefore, only two-third of chromosome I were screened to guarantee that all genes would be processed in the second screen. However, after selecting for genes that had human orthologs and rescreening in triplicate with GC and CUG strains, only 32 positive hits were selected for follow-up analysis. These hits were expected to be enriched in genes implicated in the modulation of RNA repeat toxicity. Next, we screened the 32 candidate genes as potential modifiers of expanded CUG repeat RNA toxicity. Here, we used our characterized transgenic *C. elegans* strains expressing GFP in the body wall muscle cells with a 3′ UTR encoding 123CTG repeats (123CUG) or no repeats (0CUG) (Garcia *et al*. 2014; Supplementary Fig. 1). Animals expressing RNAs with 123CUG repeats have a motility defect (Garcia *et al*. 2014). In this screen, we sought to identify genes that when inactivated would alter the motility phenotype in the 123CUG's without causing a similar phenotypic change in the 0CUG controls. Of the 32 candidates tested, 8 affected the motility of the 123CUG strain. These genes were involved in different cellular processes such as RNA binding activity or energy metabolism. The strongest phenotype was observed upon *coq-1* inactivation, which induced a severe paralysis of 2-day-old adults (Fig. 1, a and b; Supplementary Fig. 2a) and death of 123CUG's as 4-day-old adults. In addition, 123CUG animals RNAi inactivated for *coq-1* were egg-laying defective (Egl), accumulating unlaid eggs in the gonads (Supplementary Fig. 2b). Conversely, 0CUG animals downregulated for *coq-1* showed a significantly milder effect on their motility and absence of paralysis and Egl phenotypes. To test the robustness of these results, we used RNAi against *coq-1* with an independent set of transgenic strains expressing 123CUG and 0CUG repeats. These animals displayed similar phenotypes as the strains in our original screen (Supplementary Fig. 2, a and b). This shows that the potential differences in the transgenic backgrounds of these strains do not significantly contribute to the toxic phenotypes observed. These data further support the identification of *coq-1* as a suppressor of expanded CUG toxicity.

The gene *coq-1* encodes the enzyme polyprenyl synthetase, which catalyzes the first step in the biosynthetic pathway for CoQ (Tran and Clarke 2007; Supplementary Fig. 3a). Therefore, using our 2 independent strains of 123CUG and 0CUG animals, we measured CoQ levels to determine whether the expression of expanded CUGs disrupted the CoQ biosynthetic pathway. Intracellular synthesis is the major source of CoQ; however, dietary contributions can compensate for CoQ deficiencies in the biosynthetic pathway (Fernández-Ayala *et al*. 2005). For this reason, we measured CoQ levels in animals fed with the HT115  *Escherichia coli* (Fig. 1c; Supplementary Fig. 4a) or the CoQ-deficient bacterial strain GD1 (Supplementary Fig. 4b). Surprisingly, we found no difference in CoQ levels between the 123CUG animals and 0CUG controls (Fig. 1c; Supplementary Fig. 4, a and b). A small increase in CoQ was observed in strains expressing the GFP transgenes relative to the *wild-type* (*wt*) animals (Supplementary Fig. 4a) but only on animals fed with HT115  *E. coli*. Expression levels of key genes in the CoQ biosynthetic and upstream mevalonate pathways showed no significant changes common to both 123CUG strains, as 2-day-old adults. The expression changes detected were strain specific indicating allele-specific background variations and thus are unlikely to contribute to the toxic phenotypes observed (Supplementary Figs. 3a and S4, c and d). However, inactivation of *coq-1* generated a robust reduction in CoQ levels in both 123CUG and 0CUG animals (Fig. 1c) indicating that the motility phenotype observed was not due to a more efficient downregulation of *coq-1* in 123CUG animals than in 0CUG's. Although our analysis of the whole animal did not detect differences in CoQ levels between 123CUG animals and controls, previous studies using DM1 patients' blood samples described decreased CoQ levels (Siciliano *et al*. 2001). Therefore, we analyzed whether direct CoQ supplementation of 123CUG animals could revert RNA repeat toxicity. CoQ is synthesized by all aerobic organisms, with CoQ exhibiting species-specific isoforms where the isoprenoide side chain varies in length (Supplementary Fig. 3b). *C. elegans* synthesize a CoQ_9_ isoform and acquire CoQ_8_ from dietary *E. coli* (Kagan and Quinn 2000). In our assays, we used CoQ_10_, which is the predominant isoform in humans with 10 isoprene units, and has been shown to rescue defects of this biosynthetic pathway in *C. elegans* (Takahashi *et al*. 2012). 123CUG animals supplemented with CoQ_10_ showed an improvement of the motility defect (Fig. 1d). Together our data reveal that 123CUG animals phenocopy lacks CoQ without impairment of CoQ biosynthesis, supporting a role for this pathway in modulating RNA repeat toxicity.

### Expanded CUG RNA repeats disrupt mitochondrial oxidative phosphorylation

**Fig. 2.**
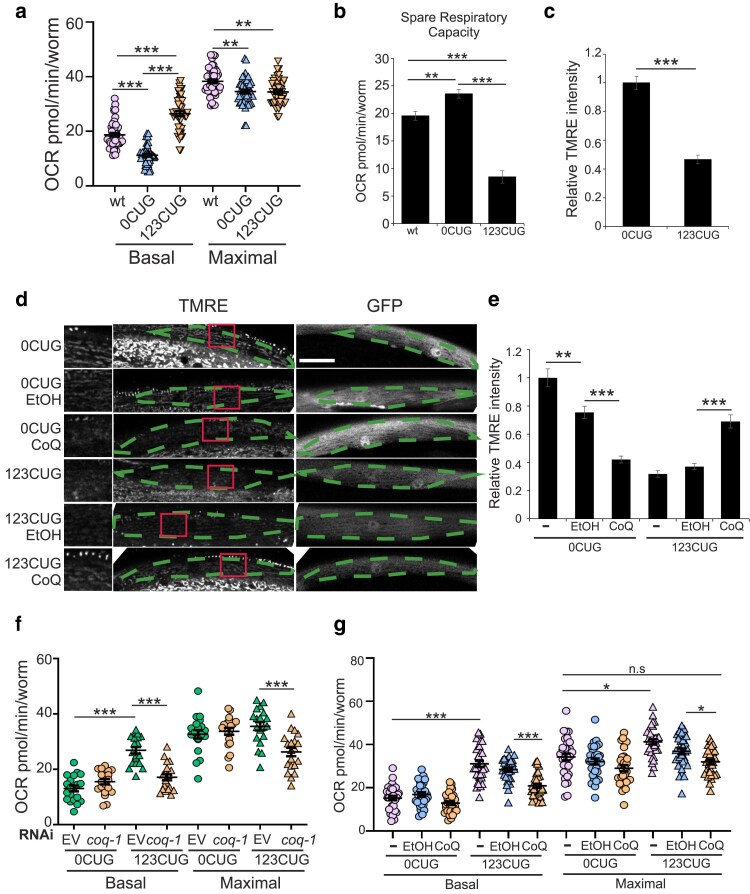
Expanded repeats cause mitochondrial dysfunction. a) Mitochondrial respiration of wt, 123CUG, and 0CUG. ***P < 0.0001, *n* = 120 animals/experiment, 4 experiments performed. b) Spare respiratory capacity of wt, 123CUG, and 0CUG's. Bars = mean ± SEM. c) Quantification of relative TMRE fluorescence in 0CUG and 123CUG body wall muscle cells. Two independent experiments performed, *n* ≥ 127 mitochondria. Bars represent mean ± SEM. d) Representative confocal images of body wall muscle with GFP reporter, and mitochondria was labeled with TMRE. Dashed lines define muscle cells. Scale bar: 25 µm. e) Quantification of relative TMRE fluorescence in 0CUG and 123CUG, with or without CoQ supplementation (7.5 µg/mL). Two independent experiments performed, *n* ≥ 50 mitochondria. Bars represent mean ± SEM. f) Effect of RNAi inactivation of *coq-1* on respiration of 0CUG and 123CUG animals. Two independent experiments performed, *n* = 100 animals/experiment. g) Effect of CoQ_10_ supplementation on respiration of 0CUG and 123CUG strains; 75 µg/mL CoQ_10_, 3 independent experiments performed *n* ≥ 100 animals/experiment. a, e, f, and g) Statistical significance was determined by 2-way ANOVA with Bonferroni correction; **P < 0.005, ***P < 0.0001. b and c) Statistical significance was determined by 2-tailed Student’s *t*-test, ***P < 0.0001.

CoQ is a highly hydrophobic lipid that is present in almost all cellular membranes but is best known for its role in the electron transport chain (ETC) in the mitochondrial inner membrane (Crane and Navas 1997; Guerra and Pagliarini 2023). CoQ plays a key role in oxidative phosphorylation (OxPhos) where it transfers electrons from complexes I and II to complex III, promoting ATP synthesis (Crane and Navas 1997; Guerra and Pagliarini 2023; Supplementary Fig. 5a). In addition to its role as a member of the ETC in the mitochondria, CoQ is also required in fatty acid oxidation, activating the uncoupling of protein function, and in uridine biosynthesis. CoQ can also function as a lipophilic antioxidant and as a membrane stabilizer (Crane and Navas 1997). CoQ's role in the ETC is essential, and changes in mitochondrial OxPhos have been described in DM1 patient fibroblasts (García-Puga *et al*. 2020); therefore, we examined whether the expression of the toxic 123CUG RNAs led to the disruption of mitochondrial function in our animals' muscle cells. OxPhos is one of the major sources of energy in muscle cells, and its disruption affects muscle function. To analyze OxPhos function, we measured mitochondrial oxygen consumption rates (OCRs) in our strains, in 2-day-old adults, and found that 123CUG animals showed a significant increase in the basal respiration rate and an unchanged maximal respiration, resulting in a substantial reduction in the spare respiratory capacity (Supplementary Fig. 5b; Fig. 2, a and b). The spare respiratory capacity reflects the animals' ability to sustain ATP production in case of an increase in energy demand. These results indicate that muscle cells in 123CUG animals have uncoupled respiration, dissipation of the potential across the mitochondrial inner membrane, and decreased capacity to respond to bioenergetic stress. To further support a link between the expression of expanded 123CUG's and the loss of mitochondrial membrane potential, we performed tetramethylrhodamine ethyl ester (TMRE) staining of our 123CUG and 0CUG strains. TMRE is a positively charged dye, which is used in the quantification of mitochondrial membrane potential changes. After staining, we analyzed the fluorescence intensity in the muscle cells of 2-day-old 0CUG and 123CUG animals (Fig. 2, c and d). Our analysis showed that TMRE fluorescence intensity was lower in 123CUG's muscle cells relative to 0CUG's (Fig. 2c), indicating loss of membrane potential. Together these data showed that expression of expanded CUGs leads to a reduction in mitochondrial membrane potential. Next, we tested whether inactivation of the CoQ pathway would further enhance 123CUG's respiration deficiency by treating our strains with *coq-1* RNAi. Inactivation of *coq-1* generated a severe decline in basal and maximal respiration in 123CUG's consistent with a collapse of the ETC function (Hill *et al*. 2012; Miyazono *et al*. 2018; Kuznetsov *et al*. 2021; Fig. 2f). However, it had no effect on the 0CUG controls. Importantly, supplementation with CoQ ameliorated these phenotypes by significantly reducing basal and maximal respiration in the 123CUG animals. Basal respiration values of 123CUG's are reduced to values closer to the 0CUG controls (Fig. 2g). To further corroborate the CoQ effects on mitochondrial function, we examined the effects of CoQ supplementation on mitochondrial membrane potential by performing TMRE dye staining of our 123CUG and 0CUG animals with and without CoQ supplementation. Analysis of TMRE staining of 2-day-old animals showed a strong increase in dye intensity in 123CUG muscle cells when supplemented with CoQ, relative to 123CUG animals without CoQ (Fig. 2e). Conversely, the 0CUG controls showed loss of dye intensity. These data suggest that CoQ supplementation contributes to ameliorate OxPhos function. However, in animals with a healthy, functional OxPhos CoQ supplementation can be deleterious to muscle cells. Together, these findings link the motility defect caused by the expression of the toxic 123CUG RNA repeats with the disruption of mitochondrial OxPhos. They further show that CoQ supplementation can improve mitochondrial function.

### Expression of expanded CUG's increases mitochondrial fragmentation

**Fig. 3.**
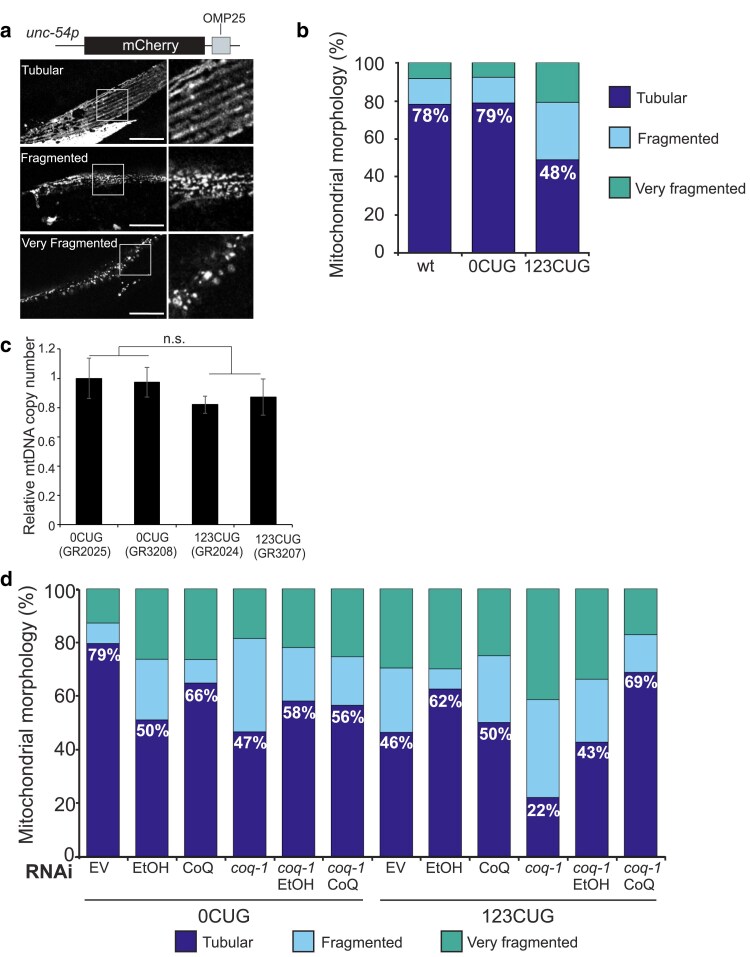
Expanded repeats disrupt mitochondrial morphology and dynamics. a) Schematic representation of mitochondrial reporter construct, and confocal images of the different mitochondrial morphologies were observed. Scale bar: 25 µm. b and d) Quantification of mitochondrial morphology of 2-day-old adults’ 123CUG and 0CUG animals. b) Without treatment; 5 independent experiments, *n* ≥ 96 mitochondria/condition. c) Relative mtDNA expression of 0CUG and 123CUG independent strains was fed on EV RNAi. Four experiments were performed with at least 3 independent biological samples. Bars represent mean ± SEM. Statistical significance was determined by 1-way ANOVA with Tukey correction. d) RNAi was inactivated for *coq-1* and supplemented with 75 µg/mL CoQ_10_. Two independent experiments were performed, *n* ≥ 39 mitochondria/condition.

There is a close interplay between mitochondrial respiratory chain function and membrane dynamics of the organelle (Takahashi *et al*. 2012). Dissipation of the mitochondrial membrane potential is associated with dramatic remodeling of organelle morphology into a fragmented state (Miyazono *et al*. 2018; Kuznetsov *et al*. 2021). To test whether mitochondrial morphology was affected by expression of the 123CUG repeats, we generated a body wall muscle–specific mitochondrial fluorescent reporter. The reporter consists of mCherry with the OMP25 outer membrane targeting sequence at the C-terminal (Fig. 3a). We crossed the reporter into both 123CUG and 0CUG strains to investigate mitochondrial morphology in 2-day-old adults. We classified mitochondrial morphology into 3 categories: tubular, fragmented, and very fragmented (Fig. 3a). Analysis of 123CUG animals showed a decrease in the abundance of tubular mitochondria and an increase in fragmented and very fragmented organelles (Fig. 3b). This shift in organelle morphology did not affect mitochondrial DNA (mtDNA) copy number (Fig. 3c; Supplementary Fig. 5c). Next, we tested whether modulation of CoQ levels could alter the mitochondrial morphology of our 123CUG animals. CoQ supplementation of 123CUG animals did not cause a significant change in morphology relative to 123CUGs on empty vector (EV) and led to a reduction in tubular mitochondria in 123CUG's when compared with animals supplemented with ethanol. Surprisingly, the ethanol alone had a striking “protective” effect on the mitochondrial morphology of 123CUG animals (Fig. 3d). The opposite effect was observed for 0CUG animals, where exposure to ethanol led to increased mitochondrial fragmentation, with CoQ supplementation causing a partial reduction of this “toxic” ethanol effect. Thus, we considered whether the ethanol supplementation may be masking the CoQ effects on mitochondrial morphology. To address this, we used a sensitized background that would allow us to distinguish the CoQ from the ethanol effects on mitochondrial morphology. We performed CoQ supplementation of 123CUG and 0CUG animals with RNAi downregulated *coq-1*. Inactivation of *coq-1* increased mitochondrial fragmentation in both 123CUG and 0CUG animals, and both 0CUG and 123CUG inactivated for *coq-1* show an increase in tubular mitochondria when ethanol is supplemented. However, CoQ supplementation specifically rescued the 123CUG's fragmentation phenotype with an increase in tubular mitochondria to levels similar to control animals, whereas it had no effect on 0CUG animals. Also, 0CUG's inactivated for *coq-1* showed a fragmentation phenotype similar to 123CUG animals on EV without ethanol. Together these data showed that the toxic phenotypes in 123CUG animals can be altered by modulating CoQ availability, worsening the phenotypes upon decrease in CoQ levels, and ameliorating them by supplementing CoQ.

Changes in mitochondrial morphology and function that result in increased organelle fragmentation and loss of membrane potential have been associated with increased production of reactive oxygen species (ROS) (Ježek *et al*. 2018). Together, these changes have been linked to a variety of human disorders including neuromuscular degenerative disorders (Chen, Zhao, *et al*. 2023). We hypothesized whether the mitochondria alterations observed in our 123CUG animals also result in increased ROS production that contributes to cellular dysfunction. To examine this, we assayed our 123CUG and 0CUG animals with *N*-acetyl-L-cysteine (NAC). NAC has a thiol group that interacts and scavenges ROS, therefore functioning as a potent antioxidant. We supplemented our 123CUG and 0CUG animals with 5 mM NAC as 2-day-old adults and tested them for changes in motility, as readout for toxicity. 123CUG animals supplemented with NAC showed an improvement of their impaired motility (Supplementary Fig. 5d). No effects were detected by supplementing 0CUG animals. These data suggest an increase in ROS production in 123CUG animals, which is reduced by the scavenger effects of NAC, contributing to a reduction in 123CUG muscle toxicity. Based on these data, we next tested our animals for induction of the mitochondrial *hsp-6* and *hsp-60* stress response genes, corresponding to the mitochondrial matrix HSP70 and HSP60 chaperones known to be upregulated by disruption of mitochondrial structure and function (Yoneda *et al*. 2004). The *hsp-6* specifically is robustly induced upon oxidative stress. Expression levels of these genes were examined for 123CUG and 0CUG animals for 2-day-old adults. No significant induction of these stress chaperones was observed for both 123CUG strains relative to the 0CUG controls (Supplementary Fig. 5e). These data suggest that although ROS is generated, there's no induction of the oxidative stress pathways that may be contributing to the toxic phenotypes.

Finally, mitochondrial dynamics is dependent on the balance between fusion and fission (van der Bliek *et al*. 2013). Because 123CUG's show a striking change in mitochondrial morphology, we sought to genetically test whether the mitochondrial dynamin-related GTPases that coordinate membrane fusion and fission modulated the 123CUG toxicity. We used RNAi to inactivate factors required for mitochondrial fission (*drp-1*) and fusion (*fzo-1* and *eat-3*) (Supplementary Fig. 5f) and assayed the motility of our transgenic CUG strains. Inactivation of *fzo-1* or *drp-1* did not cause significant changes in motility of 123CUG or 0CUG strains (Supplementary Fig. 5g). In contrast, we found that *eat-3* RNAi worsened the 123CUG's motility defect. EAT-3 regulates the inner mitochondrial membrane fusion and is known to be sensitive to uncoupled respiration (Kanazawa *et al*. 2008). These data support EAT-3 as a mild suppressor of 123CUG toxicity and supports a role for mitochondrial disruptions as key drivers of muscle dysfunction.

## Discussion

We genetically identified the CoQ biosynthetic pathway as a suppressor of expanded CUG repeat toxicity in muscle cells, using an established *C. elegans* model of DM1 dysfunction. We further linked the expression of expanded repeats with disruption of the muscle cell's metabolism, which included an increase in the requirements for mitochondrial CoQ levels and disruption of mitochondrial function and morphology with: increase in basal respiration, loss of membrane potential, decrease in spare respiratory capacity, and increase in organelle fragmentation. Here, we genetically show that this metabolic dysfunction is a key contributor to the loss of muscle function and reduced motility, associated with the expression of expanded CUGs. Moreover, we show that CoQ levels modulate expanded CUG toxic phenotypes, with CoQ supplementation ameliorating the animals' impaired motility and mitochondrial phenotypes.

The expression of expanded CUGs is known to be associated with metabolic changes (Ono *et al*. 1986; Tedeschi *et al*. 2000; Gramegna *et al*. 2018) including alteration in mitochondrial function (García-Puga *et al*. 2020); however, it is not understood how the toxic repeats cause these metabolic alterations or the functional effects of these dysfunctions in different tissues. Previous studies have suggested that changes in the OxPhos system in DM1 are associated with broad accelerated aging (Giménez-Bejarano *et al*. 2023). In support of this hypothesis, it was proposed that changes in energy metabolism precede muscle atrophy with DM1 patient samples shown to exhibit mitochondrial alterations such as changes in mitochondrial dynamics (García-Puga *et al*. 2020; Giménez-Bejarano *et al*. 2023), increased ROS generation, and decrease of CoQ levels (Siciliano *et al*. 2001; Giménez-Bejarano *et al*. 2023). Our data add to what was previously known, and we further suggest that disruption in metabolism, such as mitochondrial dysfunction, with significant changes in morphology, directly drives muscle dysfunction, with its rescue reducing impaired motility. Interestingly, our results showed normal range levels of CoQ for 123CUG animals, yet these strains behaved genetically as if they lacked CoQ with CoQ supplementation ameliorating both motility (Fig. 1d) and mitochondrial phenotypes (Figs. 2e, 2g, and 3d) of the 123CUG's. These results led us to consider that some reduction in CoQ levels may occur in the muscle cells of our animals, which is not detected by our assay. The transgenes bearing the expanded CUG repeats are exclusively expressed in the body wall muscle cells of the animals (Garcia *et al*. 2014). The muscle cell accounts for 10% of the total somatic cells in *C. elegans* hermaphrodites. Muscle cells are large and thus are able to contribute relatively large amounts of cellular material for lipidomic analysis; however, smaller variations in CoQ levels may not be detected in our whole-organism assays. Nonetheless, the genetic detection of functional effects similar to of lack of CoQ suggests that some tissue-specific reduction may be present.

Our data showed that mitochondrial morphology was affected by the addition of ethanol (EtOH) (Fig. 3d), used in our assays to solubilize CoQ. Analysis of the EtOH effects on mitochondrial morphology of our animals revealed that EtOH supplementation of 123CUG's caused an increase in tubular mitochondria, including when *coq-1* was inactivated by RNAi. An opposite effect was shown by 0CUG controls with a reduction in tubular mitochondria upon EtOH supplementation, except when inactivated for *coq-1* where, similar to 123CUG's, an increase was also present. The literature links EtOH exposure with mitochondrial impairment, affecting mitochondrial function and dynamics and promoting ROS production (Cardellach *et al*. 1992; Siggins *et al*. 2023). Our 0CUG and 123CUG data now suggest that in some metabolic disrupted states, EtOH supplementation can increase tubular mitochondria. Despite these effects, exposure to EtOH in CoQ supplementation assays had no visible effect on animal motility or respiration (Figs. 1d and 2g), supporting distinct effects of EtOH on different cellular processes. Studies have also shown that low levels of mitochondrial stress have a positive fitness effect in organisms by maintaining stress resistance and cytosolic proteostasis with aging (Labbadia *et al*. 2017). We hypothesize that exposure to EtOH in our experimental setup may contribute to a similar effect on mitochondrial morphology.

Our 123CUG animals showed a strongly reduced mitochondrial spare respiratory capacity relative to the controls (Fig. 2b). These results were consistent with a significant increase in the animals' basal respiration to levels closer to the control's maximal respiration (Fig. 2, a, f, and g). In addition, the 123CUG's weak ability to respond to FCCP (carbonyl cyanide-p-(trifluoromethoxy)phenylhydrazone) treatment, which stimulates the respiratory chain to operate at maximum capacity, indicated that the mitochondria were uncoupled, with the presence of a proton leak across the membrane. This was further corroborated by TMRE dye staining, which showed that the expression of 123CUG repeats causes a loss in membrane potential in our animals' muscle cells (Fig. 2c). Muscle cells have a high energy demand, and our results showed how RNAs bearing expanded CUG repeats, through a reduction in mitochondrial spare respiratory capacity, cause a significant decrease in the ability of mitochondria to upregulate OCR in response to sudden bioenergetic needs from the organism.

Our data also showed that variations in CoQ levels, through supplementation of CoQ or inactivation of *coq-1*, can modulate mitochondrial disruption in 123CUG animals. Because CoQ plays a central role as an electron carrier in the respiratory chain (Supplementary Fig. 5a), inactivation of *coq-1* in 123CUG animals worsened their disrupted mitochondria phenotype, leading to a strong decrease in both basal oxygen consumption and maximal respiration. A decrease in these values would suggest restoration of respiration to control's levels however, the drastic decrease to values close to the basal respiration and below the control's maximal levels, respectively (Fig. 2f), indicated the collapse of respiration and inability of the cells to respond to FCCP and increase oxygen consumption. Finally, CoQ supplementation specifically improved 123CUG's mitochondrial OxPhos with no effect on controls (Fig. 2g). This was further supported by TMRE staining data that showed that CoQ supplementation increased mitochondrial membrane potential in 123CUG muscle cells (Fig. 2e). Together, these data suggested that the CoQ effect on 123CUG respiration likely results from CoQ partially restoring the muscle's membrane potential. Conversely, CoQ supplementation of muscle cells that are not OxPhos impaired has deleterious effects on membrane potential, although these were not detectable in our respiration assays. In addition to the OxPhos effects, our data further showed that supplementation with CoQ had a striking effect on the mitochondrial morphology. Together these data support a role for CoQ in reducing mitochondrial disruption in 123CUG animals caused by the expression of the expanded RNAs. The quantification of mitochondrial respiration by OCR in our different assays was performed using the whole organism; however, the toxic expanded repeats are expressed specifically in the body wall muscle cells. For this reason, we consider that it is likely that the effects caused by the expanded repeats on mitochondrial respiration may be more pronounced than what we were able to detect. This is supported by the TMRE staining data. In addition, because these assays are performed in a multicellular organism, how metabolic dysfunction in the muscle affects neighboring tissues or if a compensatory regulatory effect is induced is not known. These would be relevant questions to address in future studies.

The disruptions in mitochondrial function and morphology observed in our 123CUG animals would also suggest the presence of increased ROS production and induction of the oxidative stress response playing a role in muscle toxicity. Increased ROS production has been detected in DM1's fibroblast cells (García-Puga *et al*. 2020) and DM1 cell models (Hasuike *et al*. 2022). Our studies also support the increased production of ROS in 123CUG's, and they further implicate them as contributors to muscle dysfunction. A reduction in ROS levels leads to a partial reduction in 123CUG's toxicity. However, ROS production in 123CUG animals does not lead to an induction of the canonical oxidative stress pathways. We hypothesize whether the chronic nature of mitochondrial dysfunction with ROS production, together with potentially low ROS levels, may be the reason why a stress response is not activated. Thus, although ROS contributes to muscle dysfunction, our data do not show the contribution of oxidative stress.

Here, we show that CoQ supplementation leads to a consistent improvement in the toxicity of 123CUG animals. Our data further support that muscle dysfunction, associated with the loss of motility observed in the 123CUG animals, results at least in part from the disruption of energy metabolism and mitochondrial function. Mitochondria are central in the regulation of the metabolic state of skeletal muscle, and their dysfunction has been associated with muscle atrophy in several diseases such as Duchenne muscular dystrophy and sarcopenia (Chen, Koh, *et al*. 2022; Chen, Ji, *et al*. 2023). Our data are in line with the results of these studies and in addition show that mitochondrial disruption significantly contributes to the progressive functional impairment of muscle cells expressing expanded CUGs. Previous research from Garcia-Puga and collaborators reported changes in mitochondrial respiration in DM1 patient fibroblasts with a decrease in both basal and maximal respiration. We hypothesize that the difference in basal and maximal respiration between our studies and the data described by Garcia-Puga *et al*. (2020) may be due to differences associated with tissue specificity. It would be of interest in future studies to examine the respiration profile of distinct tissues expressing expanded CUG repeats as these may reflect distinct metabolic profiles.

Taken together, our work shows that modulation of CoQ metabolic levels affects expanded CUG cellular toxicity with CoQ supplementation having the potential to improve the toxic phenotypes associated with mitochondrial dysfunction. Targeting the CoQ biosynthetic pathway has been previously proposed as a therapeutic approach for Duchenne muscular dystrophy (Folkers *et al*. 1985). Our studies suggest the potential of targeting the CoQ biosynthetic pathway as a complementary therapeutic option for DM1.

## Materials and methods

### *C. elegans* strains

*C. elegans* were grown following standard culture protocols (Brenner 1974), at 20°C unless otherwise indicated. The strains used were as follows: N2 Bristol as the wt strain, GR2024, GR2025, GR3207, GR3208, and GR2173. The following strains were generated for this study GAR118, GAR126, and GAR131. Genotype of strains used in this study is indicated in Supplementary Table 1. Unless otherwise stated, 0CUG and 123CUG strains used were GR2025 and GR2024, respectively.

### Plasmids and constructs

The mitochondrial outer membrane protein-25 (OMP25) sequence (Nemoto and De Camilli 1999) was cloned by PCR amplification into the *C. elegans* pPD49.26 plasmid bearing the *unc-54* body wall muscle–specific promoter and the mCherry gene. This construct was injected into wt animals at the concentration of 50 ng/µL. The PCR primers used to subclone the OMP25 into the pPD49.26 plasmid are listed in Supplementary Table 2.

### RNA interference

RNAi bacteria expressing dsRNA were grown overnight in LB media, induced the following day for 4 h with isopropylthiogalactoside (IPTG) to a final concentration of 0.5 mM, and then plated as previously described (Timmons *et al*. 2001). In *coq-1* RNAi assays, a final concentration of 6 mM IPTG was used instead. Strains to be analyzed by RNAi were synchronized by hypochlorite (NaOCl) treatment of gravid adults; their eggs were collected, plated onto RNAi plates, and left to develop. At the L4 stage, animals were transferred onto new RNAi plates containing 10 μM of 5-fluorouracil. As a negative control, we used RNAi bacteria expressing the EV L4440, unless otherwise stated.

### RNAi screens

RNAi-mediated gene inactivation was achieved through the feeding method (Kamath *et al*. 2001) using the Ahringer RNAi library (Fraser *et al*. 2000). Animals were synchronized by NaOCl bleaching, overnight hatching in M9, and then transferred onto RNAi bacteria. Briefly, in the RNA degradation screen, RNAi clones were grown overnight in 96-well 2-mL blocks containing LB with 200 μg/mL carbenicillin and then induced with 4 mM of IPTG for 3 h. The RNAi cultures were pelleted and then diluted to a 9× concentration in S-basal medium, and 10 μL of RNAi bacteria was transferred to each well in a 96-well plate. The synchronized L1s were filtered and aliquoted (30 μL) into the RNAi-containing 96-well plates (16 animals/well) and allowed to develop to the L3-stage larvae at 20°C. Animals were screened at the L3–L4 stage for the presence of fluorescence and scored from 0 to 3 based on the intensity of the GFP signal detected, with 0 having no GFP signal and 3 the strongest signal (Supplementary Fig. 1). Animals were inactivated for L4440 (EV) or *smg-2* as negative and positive controls, respectively. All candidate positives identified were rescreened twice, and gene inactivations with a score above 1.5 and a human ortholog were selected for the follow-up motility screen (32 RNAi clones). The follow-up motility screen corresponds to a toxicity screen (Supplementary Fig. 1) and was performed by: synchronizing L1s that were then transferred onto 6-cm RNAi plates and allowed to develop to 2-day-old adults (5 days after synchronization). Plates containing EV or *smg-2* RNAi bacterial clones were used as controls. Animals were analyzed for changes in locomotion, rescreened in triplicate. After rescreening, 8 RNAis were identified as altering the defective motility of 123CUG animals, without similarly affecting the 0CUG control. The RNAi clones were verified by sequencing.

### *C. elegans* motility assays

For the toxicity screen, gravid adults were synchronized by NaOCl treatment, hatched overnight in M9, and ∼150 L1 larval-stage animals were plated. Nematodes were allowed to develop to 2-day-old adults, were collected, rinsed 3 times with M9 buffer, were transferred onto 10-cm agar plates without bacteria, and let rest for 30 min prior to imaging. For each motility assay, a minimum of seven 25-s videos were recorded using an Axio Zoom.V16 microscope, at 8× magnification. For standard motility assays, gravid adults were prepared by NaOCl synchronization and plating ∼200 eggs onto seeded plates, to avoid L1 diapause that is known to alter CoQ levels. The remaining protocol was performed as indicated above. Motility data were analyzed to determine the animals' speed (μm/s) using a Matlab program developed by M. Crivaro at the Light Microscopy Unit (Institute of Biotechnology, University of Helsinki). Wilcoxon statistical analysis was used (Supplementary Table 4).

### Fluorescent and DIC (differential interference contrast) imaging

*C. elegans* images were of 2-day-old adult animals unless otherwise indicated. *C. elegans* motility images were taken using an Axio Zoom.V16 microscope, at 20× magnification. Animals were picked onto plates with 10× concentrated OP50 and imaged 2 min posttransfer. Images of Egl phenotype were taken using a Zeiss Imager.M2 microscope, at 10× objective. Nematodes to be assayed were transferred onto a 2% agarose pad on a glass slide and immobilized with 10 mM of levamisole.

Mitochondrial morphology images were obtained by mounting and immobilizing the animals expressing the mitochondrial reporter with 10 mM levamisole on a 5% agarose pad, on a glass slide. Imaging was performed using a Leica SP8 confocal microscope, with a 93× objective. The images were analyzed for morphology in a blind manner, and the different categories were similarly assigned. Total mitochondria analyzed, the percentages calculated, and statistical analysis are indicated in Supplementary Tables 3 and 4.

### Mitochondrial respiration assays

OCRs were measured using the Seahorse XFe96 Analyzer. Gravid adults were synchronized, the eggs were collected and plated on RNAi plates seeded with EV or *coq-1* RNAis, and OCR was measured when animals were 2-day-old adults. The Seahorse XF Sensor Cartridges were hydrated with XF Calibrant Solution, and these were maintained overnight at 27°C in a non-CO_2_ incubator, as described previously (Koopman *et al*. 2016). Prior to the assay, the XF Sensor Cartridge was calibrated. When assayed, the animals were picked into individual wells of a Seahorse 96-well XF Cell Culture microplate, 10 worms/well in 80 μL of M9 buffer. A minimum of 10 wells per condition was assayed. OCR measuring cycles consisted of 3 min mixing, 30 s of rest, and 3 min of analyzing oxygen consumption. For each condition assayed, four measurement were performed: basal OCR, OCR upon FCCP treatment (40 µM), and OCR upon sodium azide treatment (40 mM). Animal number, FCCP, and sodium azide concentrations were optimized for this study. Statistical analysis is indicated in Supplementary Table 4.

### CoQ supplementation assays

In CoQ supplementation assays, animals were fed EV or the CoQ-deficient GD1 strain of *E. coli* bacteria. In assays with GD1 bacteria, animals were grown on GD1 for at least 2 generations prior to the assay. CoQ supplementation was performed by dissolving the CoQ_10_ in ethanol and adding it to the agar at the desired concentration. Ethanol was used as a control at the same concentration (v/v) as CoQ. In motility assays, the plates were supplemented with 150 μg/mL of CoQ_10_ (Sigma). Motility analysis was performed as described. CoQ_10_ was used in mitochondrial membrane potential assays at a concentration of 75 g/mL. Respiration and mitochondrial assays on EV or *coq-1* RNAi were supplemented with 75 μg/mL of CoQ_10_. Imaging and respiration analysis were performed as described.

### NAC treatment

NAC (Sigma) was dissolved in dH_2_O and added to RNAi plates to a final concentration of 5 mM. Plates were seeded with EV. Motility assay was performed as described.

### Quantitative RT-PCR

Animals were collected (≈2,000) from 3 biological replicas as 2-day-old adults and frozen in liquid nitrogen. Total RNA was extracted using TRIzol Reagent (Ambion), followed by chloroform extraction and isopropanol precipitation. cDNA was synthesized from 250 ng RNA using QuantiTect Reverse Transcription Kit (Qiagen), and qRT-PCR reactions were performed with SYBR Green reagent (Roche) using Lightcycler 480 (Roche). qRT-PCR data were normalized to *cdc-42* and *pmp-3* gene expressions. The 2^−ΔΔ*ct*^ method was used for comparing relative levels of mRNAs. Primers and statistical analysis are listed in Supplementary Tables 2 and 4, respectively.

### Mitochondrial copy number

Mitochondrial copy number was determined by qPCR of 2-day-old adult samples, as described previously (Bratic *et al*. 2010). Animals were collected (≈2,000) in lysis buffer (50 mM KCl, 10 mM Tris-HCl ph 8.3, 2.5 mM MgCl_2_, 0.45% NP-40, 0.45% Tween-20, 0.01% gelatin, and 60 μg/mL protease K), incubated at 60°C for 2 h, followed by 15 min at 95°C. Samples were used for qRT-PCR performed with SYBR Green reagent (Roche) using Lightcycler 480 (Roche). Expression data were normalized to *act-1* gene expression levels. Primers and statistical analysis are indicated in Supplementary Tables 2 and 4, respectively.

### TMRE staining

Staining was performed as described previously (Wang *et al*. 2016; Wei and Ruvkun 2020) with some modifications. Briefly, animals were synchronized and grown on EV. At the L4 stage, animals were transferred to plates with 5′-fluorouracil. On the day prior to the imaging (1-day adult stage), animals were transferred to plates containing 3 μM of TMRE (Sigma, 87917). On the day of the assay, animals were transferred to 1.5-mL Eppendorf tubes and washed 3× with M9. Strains were then transferred to new plates without TMRE and recovered for 1 h. Imaging was performed as described previously.

### Sample preparation for lipid analysis

Three biological samples were collected (≈1,000 animals/sample) as 2-day-old adults, transferred to 2-mL polypropylene tubes with 250 μL of lipid lysis buffer (20 mM Tris-HCl pH 7.4, 100 mM NaCl, 0.5 mM EDTA, and 5% glycerol), and kept on ice for 15 min. Samples were homogenized using a Precellys 24 homogenizer at 6,400 rpm, twice for 10 s with 5-s interval. This homogenization procedure was repeated twice, and samples were kept on ice between homogenizations.

### Lipid extraction

Lipid extraction was performed as described previously (Pellegrino *et al*. 2014) with some modifications. One milliliter of a solution of methanol:methyl tertiary-butyl ether:chloroform (MMC) 1.33:1:1 (v/v/v) was added to 20 µL of the worm lysate. The MMC sample mixture was fortified with the SPLASH mix of internal standards (Avanti Lipids). The samples were vortexed briefly and then kept shaking continuously in a Thermomixer (Eppendorf) at 25°C, for 30 min at 950 rpm. Next, samples were centrifuged for 10 min, at 16,000*g*, and at 25°C to obtain protein precipitation. The single-phase supernatant was collected, dried under N_2_, and stored at −20°C until analysis. Before analysis, the dried lipid samples were redissolved in 100-µL MeOH:isoproanol (1:1).

### Liquid chromatography mass spectrometry conditions

Liquid chromatography was done as described previously (Cajka and Fiehn 2016) with some modifications. Lipids were separated using C18 reverse phase chromatography. Vanquish LC pump (Thermo Scientific) was used with the following mobile phases; (A) acetonitrile:water (6:4) with 10 mM ammonium acetate and 0.1% formic acid and (B) isopropanol:acetonitrile (9:1) with 10 mM ammonium acetate and 0.1% formic acid. The Acquity BEH column (Waters) with the dimensions 100 mm × 2.1 mm × 1.7 µm (length × internal diameter × particle diameter) was used. The following gradient was used with a flow rate of 0.6 mL/min; 0.0–2.0 min (isocratic 30% B), 2.0–2.5 min (ramp 30–48% B), 2.5–11 min (ramp 48–82% B), 11–11.5 min (ramp 82–99%), 11.5–12 min (isocratic 100% B), 12.0–12.1 min (ramp 100–30% B) and 12.1–15 min (isocratic 30% B).

The liquid chromatography was coupled to a hybrid quadrupole-orbitrap mass spectrometer (Q-Exactive HFx, Thermo Scientific). A full scan acquisition in negative and positive ESI (electrospray ionization) was used, scanning from 200 to 2,000 m/z at a resolution of 120,000 and AGC Target 1e6 and max injection time of 200 ms. Data-dependent scans (top 10) were acquired using normalized collision energies of 20, 30, and 50 and a resolution of 15,000 and AGC target of 1e5.

### Ubiquinone identification

Identification of the quinone lipids was achieved using 4 criteria: (1) high accuracy and resolution with an accuracy within m/z within 5 ppm shift from the predicted mass and a resolving power of 70,000 at 200 m/z. (2) Isotopic pattern fitting to expected isotopic distribution. (3) Comparison of the retention time with an in-house database and (4) fragmentation pattern correspondence to an in-house experimentally validated lipid fragmentation database. Quantification was done using single point calibration by comparing the area under the peak of different quinones with the area under the peak of SPLASH internal standard closed in time to the area under the peak of the internal standard and then normalized to protein concentration. Mass spectrometric data analysis was performed in TraceFinder software 4.1 (Thermo Scientific) for peak picking, annotation ,and matching to the in-house fragmentation database.

### Statistical analysis

Statistical analysis of motility assays was performed using Wilcoxon statistical test with the Bonferroni correction. A minimum of 2 independent experiments were performed per gene and condition tested. Statistical analysis of mitochondrial respiration was performed using 2-tailed Student's *t*-test or 2-way ANOVA statistical test, if different conditions or genes were analyzed. The Bonferroni correction was used in both statistical tests. A minimum of 2 independent experiments were performed in mitochondrial respiration assays, per gene or condition tested. Statistical test of qRT-PCR, spare respiratory capacity, and CoQ_9_ lipid levels were determined using 2-tailed Student's *t*-test. Statistical analysis of mtDNA was performed using 1-way ANOVA with Tukey correction.

## Supplementary Material

iyae208_Supplementary_Data

## Data Availability

Strains and plasmids are available upon request. The authors affirm that all data necessary for confirming the conclusions of the article are present within the article, figures, and tables. Supplemental material available at GENETICS online.
