# Supplementary material for: Coenzyme Q improves mitochondrial and muscle dysfunction caused by CUG expanded repeats in Caenorhabditis elegans
Source: Genetics. 2024 Dec 27;229(2):iyae208. doi: 10.1093/genetics/iyae208 (PMC12230797; doi:10.1093/genetics/iyae208)
Supplement: iyae208_Supplementary_Data [file iyae208_supplementary_data.zip › Supplemental_Table_Legends.docx]

**Supplementary Tables Legends**

Table S1: List of all strains used and generated in this study.

Table S2: Oligonucleotide sequences used for RT-PCR analysis and qPCR.

Table S3: Mitochondrial fragmentation data used to generate graphs.

Table S4: Statistical analysis data of the assays performed in this study
