# Supplementary figures and images for: Coenzyme Q improves mitochondrial and muscle dysfunction caused by CUG expanded repeats in Caenorhabditis elegans
Source: Genetics. 2024 Dec 27;229(2):iyae208. doi: 10.1093/genetics/iyae208 (PMC12230797; doi:10.1093/genetics/iyae208)

# Figure S1

## 1<sup>st</sup> screen - RNA degradation screen

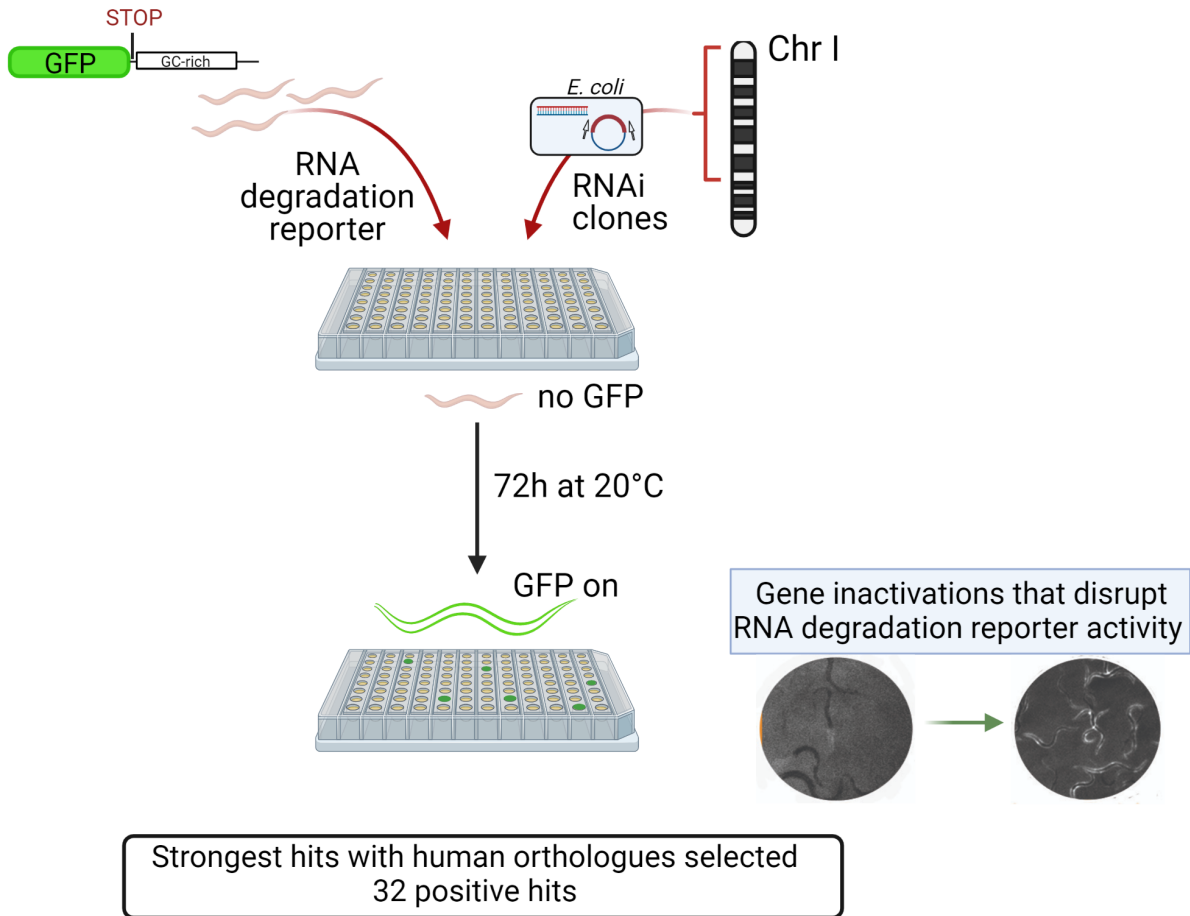

## 2<sup>nd</sup> screen - RNA toxicity screen

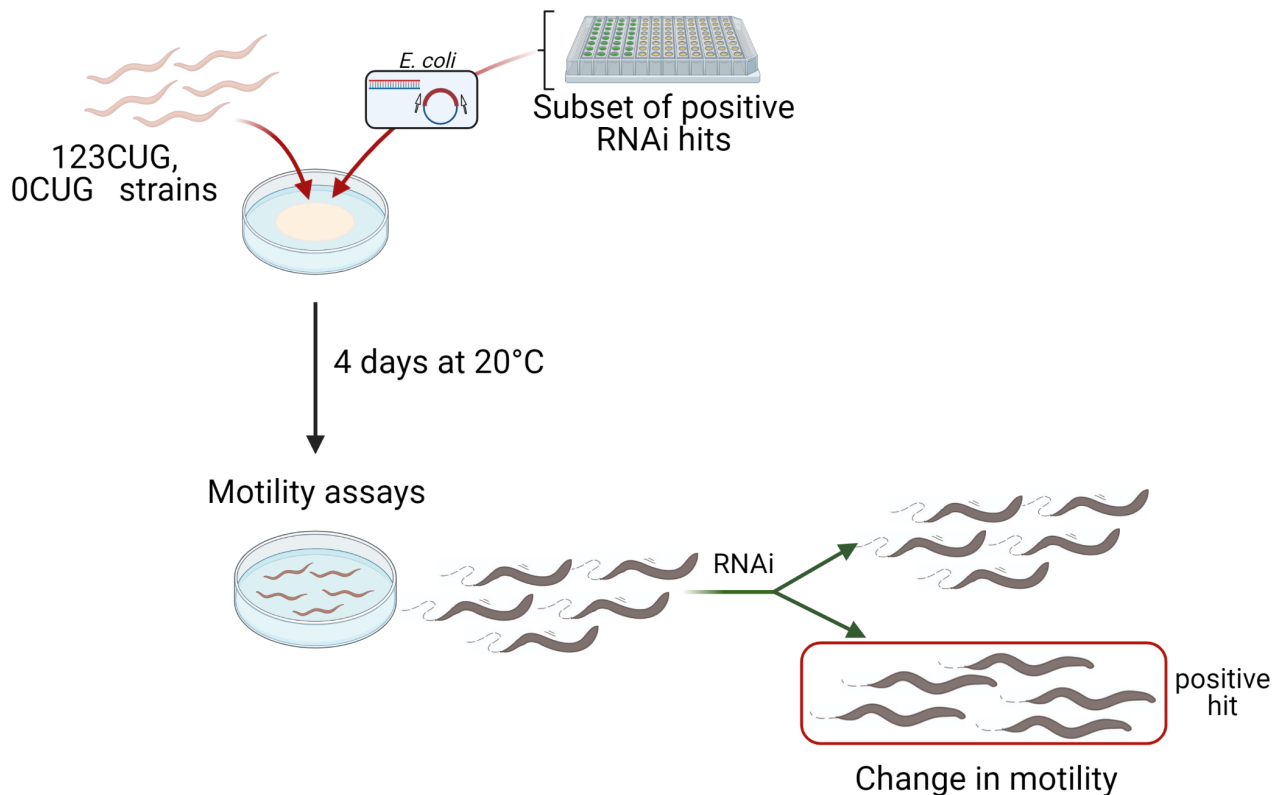

Supplement: iyae208_Supplementary_Data [file iyae208_supplementary_data.zip › Figure_S1_GENETICS-2024-307444.pdf]

# Figure S2

a

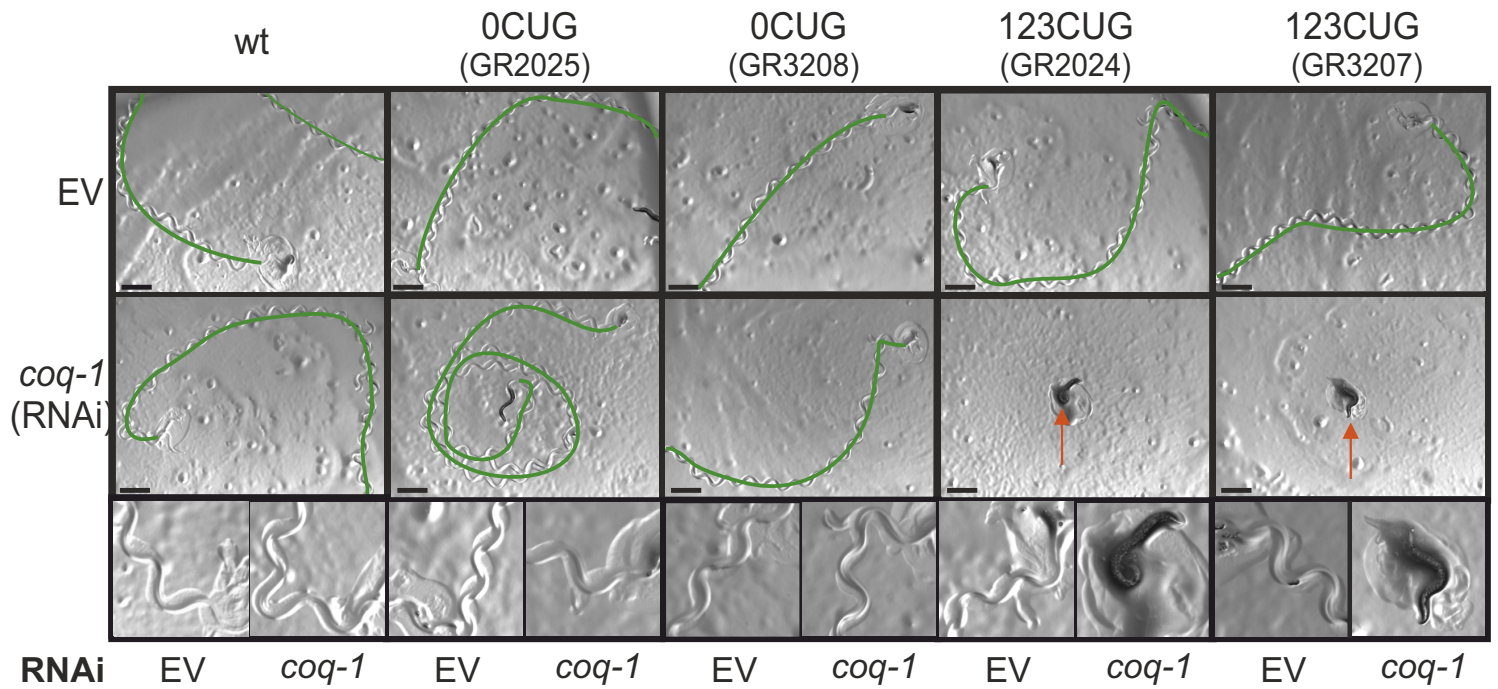

b

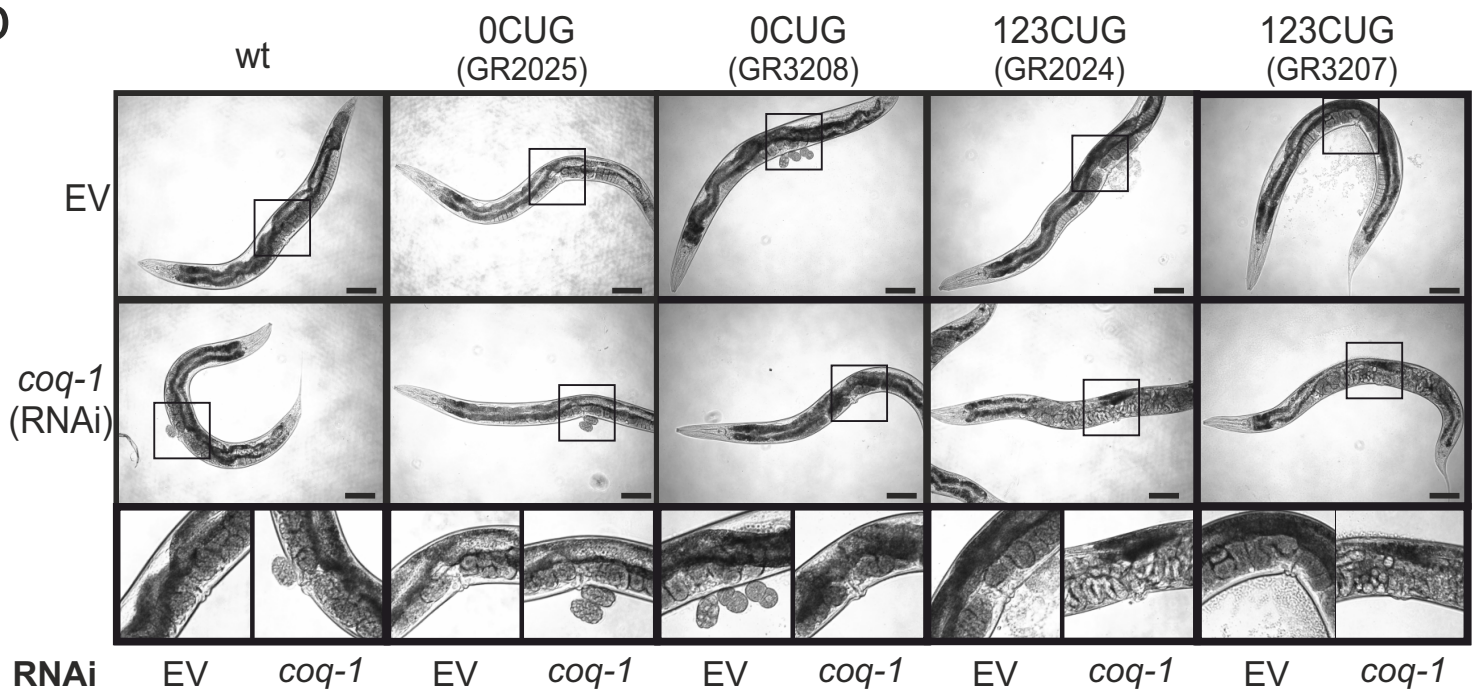

Supplement: iyae208_Supplementary_Data [file iyae208_supplementary_data.zip › Figure_S2_GENETICS-2024-307444.pdf]

Figure S3

a

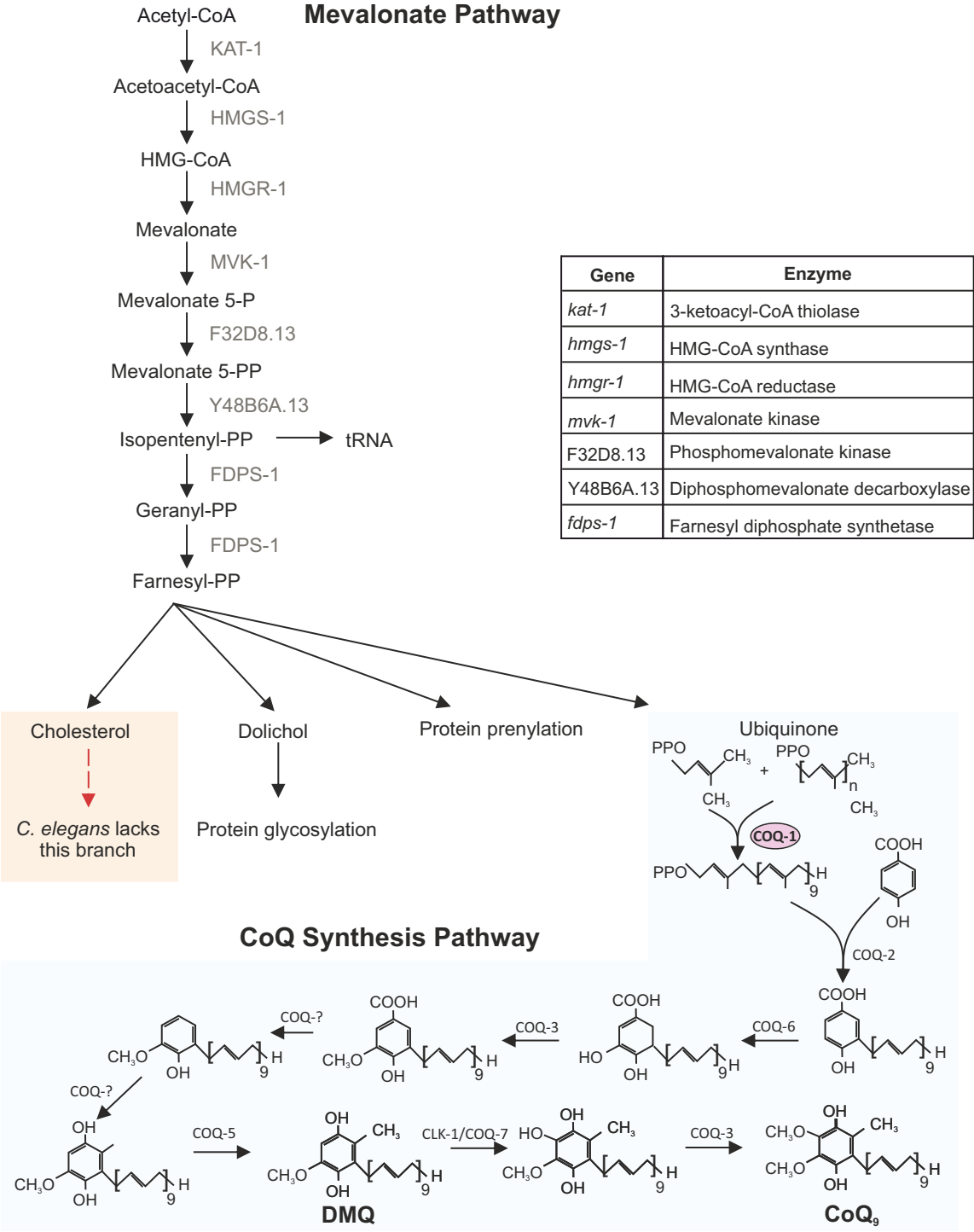

b

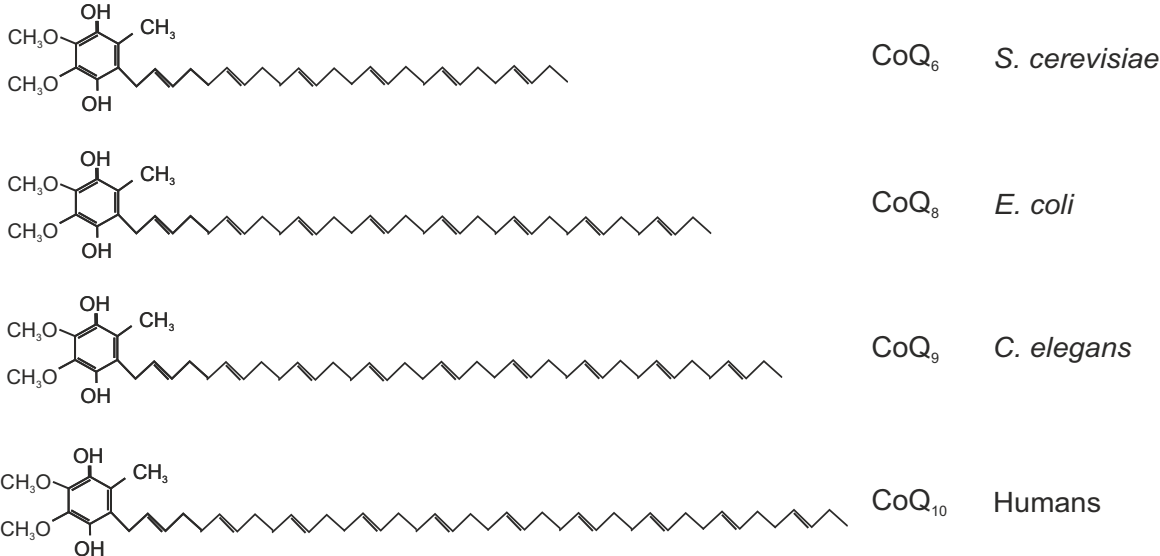

Supplement: iyae208_Supplementary_Data [file iyae208_supplementary_data.zip › Figure_S3_GENETICS-2024-307444.pdf]

Figure S4

a

Animals fed on EV

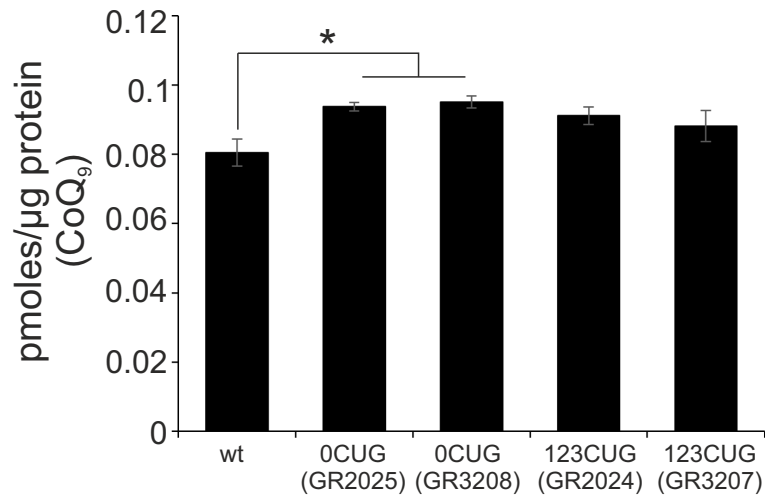

b

Animals fed on GD1

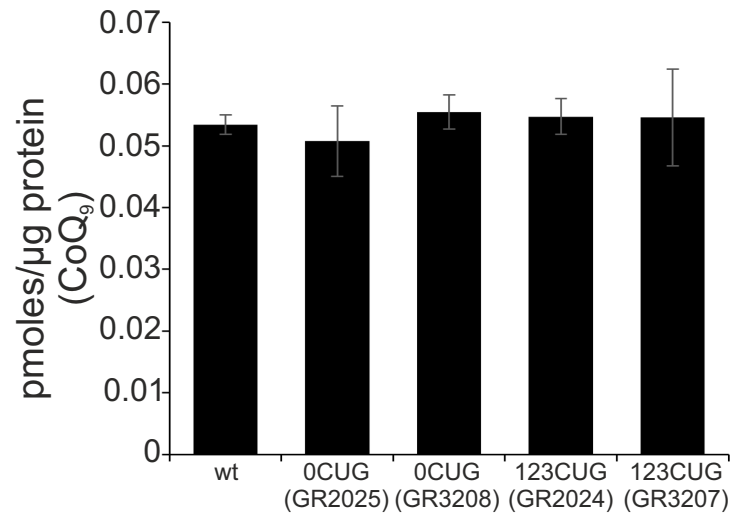

c

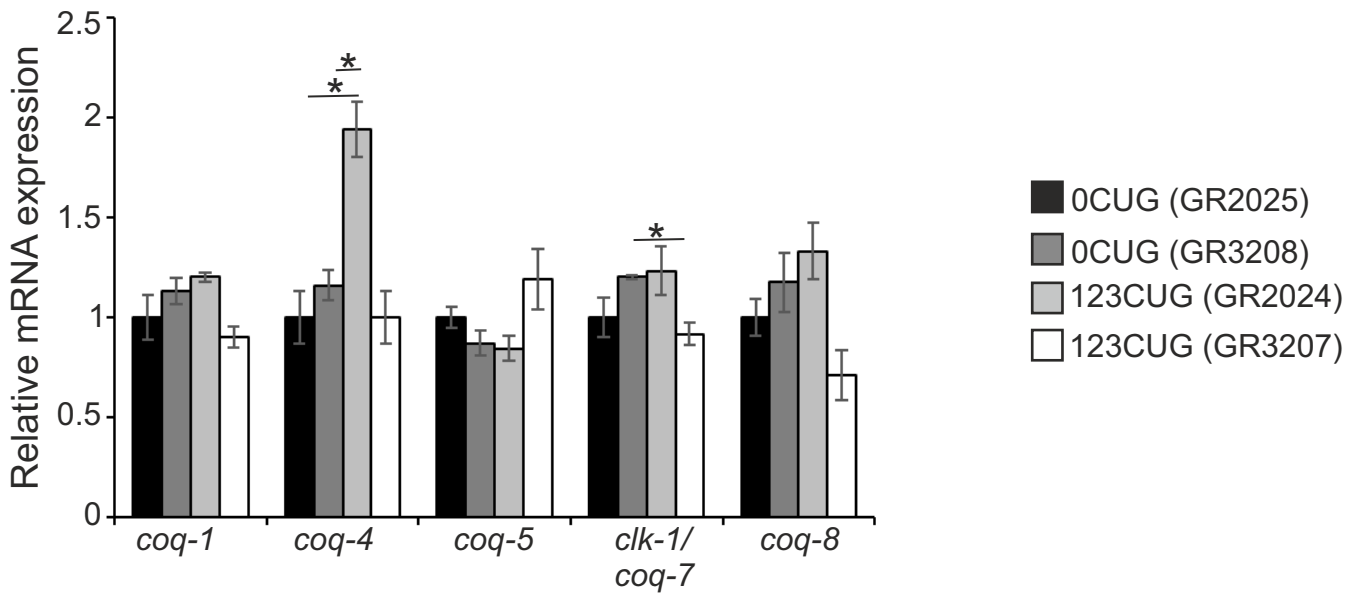

d

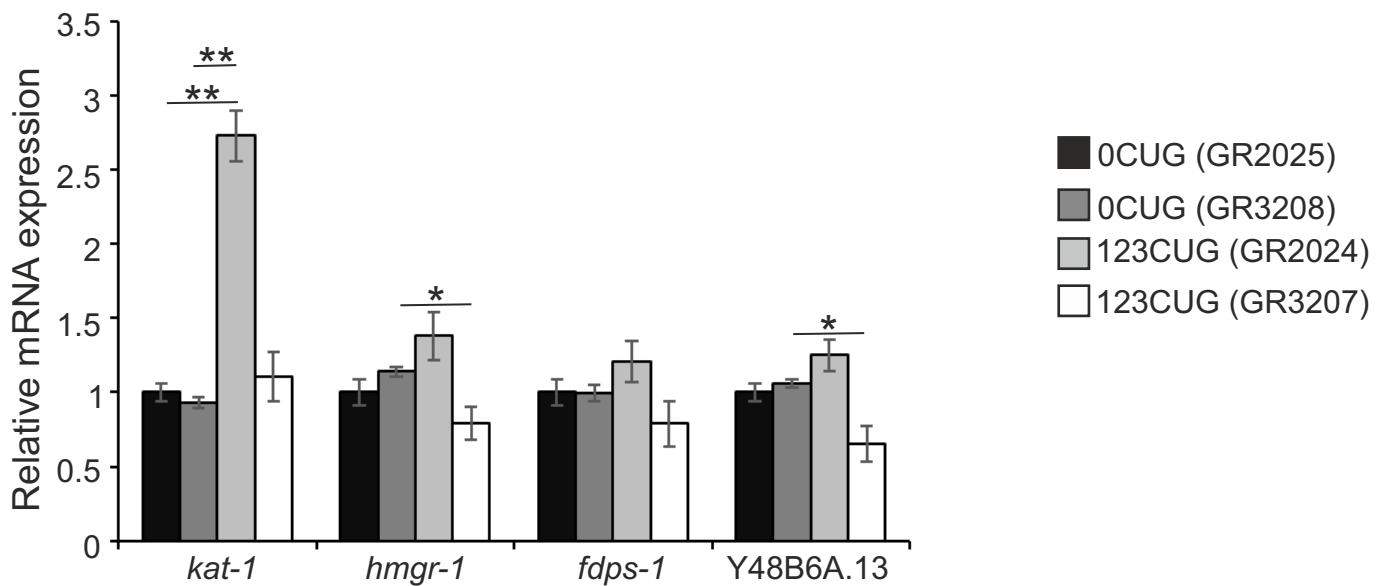

Supplement: iyae208_Supplementary_Data [file iyae208_supplementary_data.zip › Figure_S4_GENETICS-2024-307444.pdf]

# Figure S5

## Electron transport chain

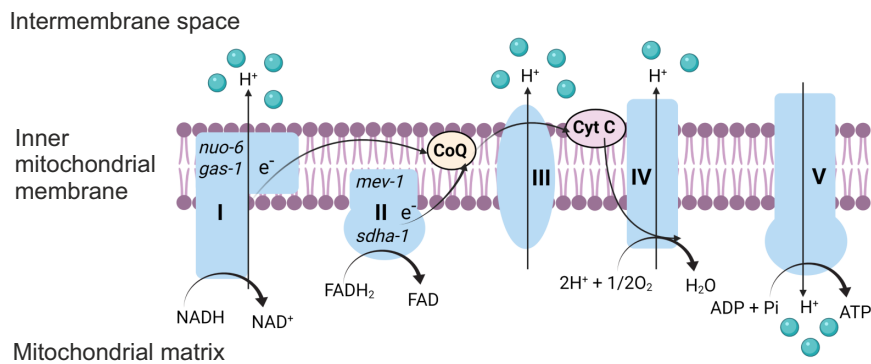

**b**

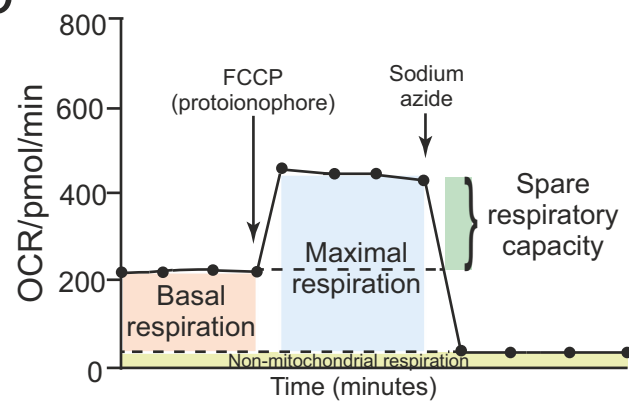

**c**

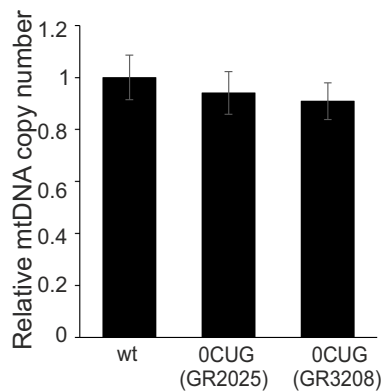

**d**

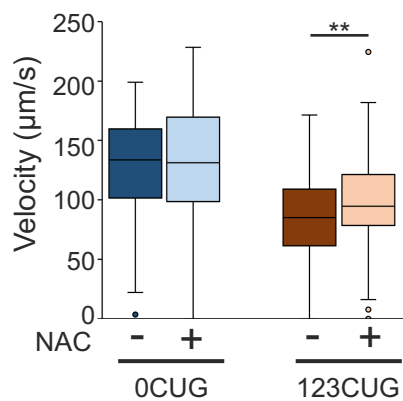

**e**

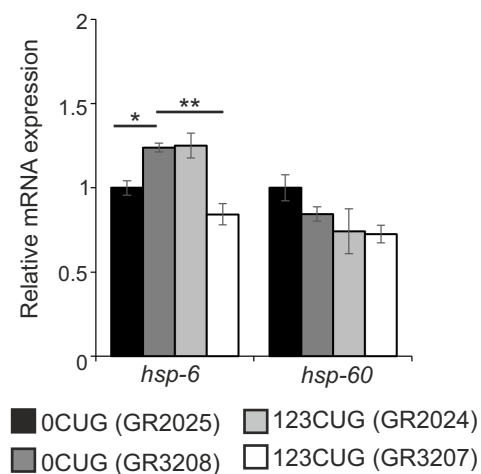

**f**

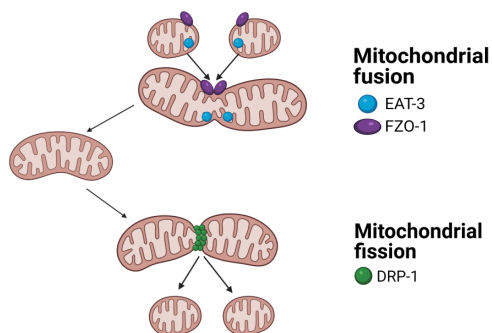

**g**

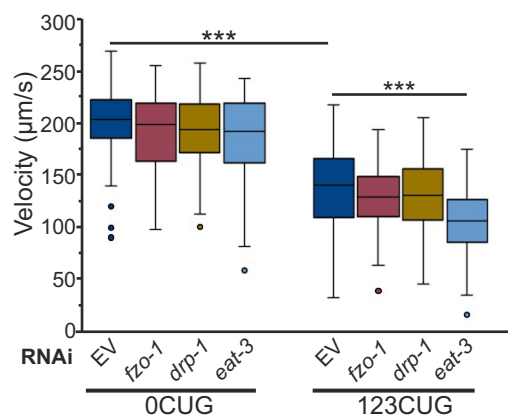

Supplement: iyae208_Supplementary_Data [file iyae208_supplementary_data.zip › Figure_S5_GENETICS-2024-307444.pdf]
