# Supplementary material for: Coenzyme Q improves mitochondrial and muscle dysfunction caused by CUG expanded repeats in Caenorhabditis elegans
Source: Genetics. 2024 Dec 27;229(2):iyae208. doi: 10.1093/genetics/iyae208 (PMC12230797; doi:10.1093/genetics/iyae208)
Supplement: iyae208_Supplementary_Data [file iyae208_supplementary_data.zip › Supplemental_Figure_Legends_GENETICS-2024-307444.doc]

**Supplementary Figures Legends**

Figure S1: Diagram of RNAi screens for the identification of modifiers of RNA repeat toxicity.

Two sequential RNAi screens were performed to identify modifiers of CUG-RNA repeat toxicity. 1^st^ screen: a *C. elegans* RNA reporter strain (GR2173) expressing a GFP with a GC-rich 3’UTR was used to analyze by RNAi two-thirds of Chr I and identify genes that function as potential modifiers of RNA degradation pathways. Genes were selected as positives if, when inactivated, interfered with the degradation of the GC-rich reporter transcript, leading GR2173 animals to exhibit a GFP fluorescent signal. 2^nd^ screen: the strongest hits with human orthologues (32 clones) identified from the first screen were selected. These RNAi clones were used in motility assays with 123CUG and 0CUG strains to identify modifiers of CUG expanded toxicity. Genes were identified as positives for their ability, when inactivated, to modulate the motility defect of 123CUG animals without similarly affecting the 0CUG strain. For the detailed motility experimental procedure, see Methods. Image created with BioRender.com.

Figure S2: Phenotypes observed upon inactivation of *coq-1* are repeat dependent.

(A, B) Representative DIC images of 0CUG (GR2025 and GR3208) and 123CUG (GR2024 and GR3207) strains RNAi inactivated for EV and *coq-1*. (A) *coq-1* inactivation causes paralysis of 123CUG strains but does not affect the controls. Green line indicates visible tracks left by the animals, and the arrow indicates the paralyzed animals. Scale bar, 1mm. (B) Presence of Egl phenotype in 123CUG when *coq-1* is inactivated. Scale bar, 100µm.

Figure S3: Diagram of CoQ biosynthesis.

(A) CoQ biosynthesis includes the mevalonate pathway and the CoQ synthesizing pathway. The mevalonate pathway catalyzes the synthesis of essential intermediates for tRNA modification and converts acetyl-CoA and acetoacetyl-CoA to farnesyl diphosphate, a precursor of sterol isoprenoids such as cholesterol. Additionally, farnesyl diphosphate feeds into cascades that synthesize non-sterol isoprenoids, such as ubiquinone, as well as intermediates for protein prenylation. In *C. elegans*, the sterol synthesis branch involved in the synthesis of cholesterol is absent. (B) The CoQ polyprenyl tail length is species-specific and it’s determined by the number of isoprene units. The subscript in the designation CoQn refers to the number of isoprene units.

Figure S4: Expression of CoQ biosynthetic factors is not affected in 123CUG animals.

(A-B) CoQ_9_ lipid levels of wt, 0CUG (GR2025 and GR3208) and 123CUG (GR2024 and GR3207) strains fed on (A) OP50 or (B) GD1 bacteria. (C-D) Analysis of mRNA relative expression levels of genes from (C) CoQ synthesis and (D) mevalonate pathways. Bars represent mean ± S.E.M of three biological replicates, each with three technical replicates. P-value determined by two-tailed Student’s *t*-test, *p<0.05, **p<0.005.

Figure S5: Mitochondrial dynamics is altered in 123CUG animals.

(A) Schematic representation of the electron transport chain (created with BioRender.com). During mitochondrial respiration, the electrons resulting from the conversion of NADH to NAD+ (complex I) and FADH_2_ to FAD (complex II) are transferred by CoQ to complex III. Simultaneously, complexes I, III and IV pump protons present in the mitochondrial matrix into the intermembrane space. These mechanisms produce a proton gradient that is used to generate ATP through complex V. (B) Representative oxygen consumption rates (OCR) profile of mitochondrial function assay. The mitochondrial uncoupler FCCP and the inhibitor sodium azide are used to determine maximal respiration and oxygen consumption from non-mitochondrial sites, respectively. (C) Relative mtDNA expression of wt and 0CUG independent strains fed on empty vector RNAi. Four experiments were performed with three independent biological samples. Bars represent mean ± S.E.M. Statistical significance determined by two-tailed Student’s *t*-test. (D) Velocity of 0CUG and 123CUG animals upon supplementation of 5mM NAC. (E) Analysis of mRNA relative expression of genes involved in oxidative response. Bars represent mean ± S.E.M. (F) Schematic representation of mitochondrial dynamics (created with BioRender.com). (G) Velocity of 0CUG and 123CUG strains upon RNAi inactivation of mitochondrial dynamics regulatory genes. ***p<0.0001. Two independent experiments performed, n≥90 animals/ condition. (D, G) Statistical significance was determined by Wilcoxon statistical test, with Bonferroni correction; Box: 25^th^ to 75^th^ percentile; whiskers: 1.5 * interquartile range; line in the box: median; dots: outliers.
